# Screening and Brief Interventions for Hazardous and Harmful Alcohol Use among University Students in South Africa: Results from a Randomized Controlled Trial

**DOI:** 10.3390/ijerph10052043

**Published:** 2013-05-21

**Authors:** Supa Pengpid, Karl Peltzer, Hendry van der Heever, Linda Skaal

**Affiliations:** 1 Department of Health System Management and Policy, University of Limpopo (MEDUNSA Campus), Pretoria 0424, South Africa; E-Mails: supaprom@yahoo.com (S.P.); hvdheever@gmail.com (H.H.); lskaal@yahoo.com (L.S.); 2 ASEAN Institute for Health Development, Madidol University, Salaya, Phutthamonthon, Nakhonpathom 73170, Thailand; 3 HIV, AIDS, TB, and STIs (HAST), Human Sciences Research Council (HSRC), Pretoria 0001, South Africa; 4 Department of Psychology, University of Limpopo, Turfloop, Sovenga 0727, South Africa

**Keywords:** alcohol misuse, associated factors, brief intervention trial, university students, South Africa

## Abstract

The aim of this study was to assess the effectiveness of Screening and Brief Intervention (SBI) for alcohol problems among university students in South Africa. The study design for this efficacy study is a randomized controlled trial with 6- and 12-month follow-ups to examine the effects of a brief alcohol intervention to reduce alcohol use by hazardous and harmful drinkers in a university setting. The unit of randomization is the individual university student identified as a hazardous or harmful drinker attending public recruitment venues in a university campus. University students were screened for alcohol problems, and those identified as hazardous or harmful drinkers were randomized into an experimental or control group. The experimental group received one brief counseling session on alcohol risk reduction, while the control group received a health education leaflet. Results indicate that of the 722 screened for alcohol and who agreed to participate in the trial 152 (21.1%) tested positive for the Alcohol Use Disorder Identification Test (AUDIT) (score 8 or more). Among the 147 (96.7%) university students who also attended the 12-month follow-up session, the intervention effect on the AUDIT score was −1.5, which was statistically significant (*P* = 0.009). Further, the depression scores marginally significantly decreased over time across treatment groups, while other substance use (tobacco and cannabis use), self-rated health status and Posttraumatic Stress Disorder (PTSD) scores did not change over time across treatment groups. The study provides evidence of effective brief intervention by assistant nurses with hazardous and harmful drinkers in a university setting in South Africa. The short duration of the brief intervention makes it a realistic candidate for use in a university setting.

## 1. Introduction

The use of alcohol in South Africa is among the highest in Africa, with a total adult *per capita* consumption of 9.5 L pure alcohol per year [1]. High hazardous or harmful alcohol use has been found among alcohol users in South Africa [2,3], with a *per capita* consumption of 34.9 L pure alcohol per year (men 39.6 L, women 23.8 L) among people that drink alcohol [1]. Hazardous drinking is defined as a quantity or pattern of alcohol consumption that places patients at risk for adverse health events, while harmful drinking is defined as alcohol consumption that results in adverse events (e.g., physical or psychological harm) [4]. 

Few studies in Africa and South Africa have found a high prevalence of hazardous or harmful alcohol use among university students, e.g., in Malawi 54.1% among males and 16.5% among females [5]. Young and de Klerk [6] studied the patterns of alcohol usage on a South African university campus in 2008, and found that using a cut off of 8 on the Alcohol Use Disorder Identification Test (AUDIT) 33.4% were hazardous drinkers (AUDIT 8–15), 7.8% harmful drinkers (AUDIT 16–19) and 9.0% probable alcohol dependent (AUDIT 20–40). In a study on the same university campus two years later found using a cut off of 6 for women and 8 for men 57.8% were found to be hazardous or harmful drinkers (men: 57.9% women: 57.8%) [7]. This pattern of alcohol consumption by university students may be typical and a cause of concern, as these youths are starting a new period of life, often leaving their homes for the first time, and experiencing freedom along with the use of alcohol and other drugs [8,9]. Social, attitudes and health factors associated with alcohol consumption or problem drinking in university students have been identified as social factors [10] such as (peer) drinking norms [11,12,13,14,15], attitudes towards alcohol use, perceived susceptibility of alcohol use, perceived self-efficacy [16], other substance use such as tobacco use [12,17] and cannabis use [18], depression [17,19,20], and posttraumatic stress [21].

Screening and brief alcohol intervention has been found an effective preventive method to reduce hazardous or harmful alcohol use, including in university settings [22,23]. Brief interventions for hazardous or harmful alcohol users may include assessing drinking patterns, giving personalized feedback, dealing with resistance and ambivalence, aiming at reduced alcohol use or abstinence, reviewing a client-centred workbook and having reinforcement visits [24]. A number of randomized controlled trials have shown [22,23] including two among university students in middle incomes countries (Brazil) [9] and Thailand [25] that, in comparison with controls, hazardous and harmful drinkers receiving brief intervention will significantly reduce alcohol consumption. There is a lack of studies on screening and brief intervention of alcohol problems in university settings in low and middle income countries. Therefore, the aim of this study was to assess the effectiveness of Screening and Brief Intervention (SBI) for alcohol problems among university students in South Africa using a randomized controlled trial design.

## 2. Methodology

### 2.1. Design

The study design for this efficacy study is a randomized controlled trial with 6- and 12-month follow-ups to examine the effects of a brief alcohol intervention to reduce alcohol use by problem drinkers among university students. The unit of randomization was the individual student identified as a hazardous or harmful alcohol user attending one university in the Gauteng Province in South Africa. Students consented to participate were informed that they could either receive or not get a brief intervention.

### 2.2. Study Population and Participants

The sample included students of one university in Gauteng Province. Students were screened for alcohol problems, and those identified as hazardous or harmful drinkers were randomized into an experimental or control group. The experimental group received one brief counselling session on alcohol risk reduction, while the control group received a health education leaflet. 

#### 2.2.1. Principles for Recruitment

##### Inclusion criteria

University students (males and females) 18 years and above, who visited public recruitment venues at the university campus and who scored as risk drinkers (*i.e.*, 8 or more on the Alcohol Use Disorder Identification Test (AUDIT) questionnaire) were included in this study.

##### Exclusion criteria

University students with a score of less than 8 on the AUDIT questionnaire, those who are pregnant, and those who are already under alcohol treatment, were excluded.

#### 2.2.2. Participant Randomization

After baseline assessment, each student was randomized to either a control or a brief intervention group. Students were randomized using sequentially numbered opaque sealed envelopes prepared according to a computer-generated (prepared using Stata version 10) randomization allocation sequence. This was carried out separately by an off-side data management group. After randomization interventionists were instructed to implement the brief intervention.

##### Blinding

Research assistant nurses and university students were not blind to their intervention. However, to protect against information biases in the reporting of alcohol use behaviour, the data collection team who assessed the outcomes were blind to the client’s status as intervention arm.

#### 2.2.3. Procedure

Universal screening of all presenting university students at the public recruitment venues was used whereby all consecutive students visiting the public venues were screened for alcohol problems and randomized into an intervention or control group. A student health promotion study had previously been advertised on the campus for university students. Research assistant 1 asked for consent from students attending the public recruitment venues to participate in the study, *i.e.*, do a baseline assessment using the AUDIT questionnaire. Research assistant 1 was not involved in delivering treatment. Research assistant nurse 2 scored the results of the alcohol test section of the questionnaire. University students who scored 8 or more on the AUDIT questionnaire after the screening (risky drinkers) were being included in this study. Research assistant nurse 2 implemented the randomization to intervention or control arms. Research assistant nurse 2 carried out the intervention for all the participants, after which they were followed up at 6 months and 12 months, and assessments were done by Research assistant 1, who was blinded to the intervention allocation of the participants. 

In the event of a dropout, at least six individual attempts were made to contact patients by telephone and letter. Even if a follow-up contact was not successful at 6 months, further attempts were made at 12 months. Sampling occurred throughout the day when the university was in session over a 3-month period. Participants received 80 South African Rands (10 US$) for the time of participation in all assessments at the time of the completion of the second follow-up assessment. Questionnaires were administered in English at baseline, 6 and 12 months follow-up visits; the full questionnaire at baseline and 12 months follow-up, while at 6 months only the AUDIT was administered. We received ethical approval from the Medunsa Research and Ethics Committee (Project number: MREC/H/43/2011:IR). The university management of the study university also provided approval for this study. The study was conducted from August 2011 to November 2012.

#### 2.2.4. Interventions

##### Control arm: provision of health education leaflet

Participants randomized to this group received feedback on the initial alcohol screening. They were provided with a health education leaflet on responsible drinking.

##### Experimental arm: brief intervention

Participants who were randomized onto the brief intervention arm receive personalized feedback on their AUDIT results, a health education leaflet, simple advice plus brief counselling about reducing excessive drinking, during a one session 20 min intervention [25]. The major steps of brief counselling were: (1) To identify any alcohol related problems mentioned in the interview, (2) To introduce the sensible drinking leaflet, emphasize the idea of sensible limits, and make sure that patients realize that they are in the medium-risk drinking category, (3) To work through the first 3 sections of the problem solving manual while mentioning the value of reviewing the other sections, (4) To describe drinking diary cards, (5) To identify a helper, and (6) To mention the 6 and 12 months follow-up assessments [26]. The Information-Motivation-Behavioural Skills (IMB) Model was used to guide the alcohol reduction intervention. The IMB model [27,28,29] proposes that information about alcohol misuse and methods of reducing and preventing harmful and/or hazardous drinking is a necessary precursor to a risk reduction. Motivation to change, however, also directly affects whether one acts on information about risk and risk reduction. Finally, the IMB model holds that behavioural skills related to preventive actions represent a final common pathway for information and motivation to result in alcohol risk behaviour change. The IMB model posits that information and motivation activate behavioural skills to ultimately enact risk reduction behaviours. The IMB model also shows that information or motivation alone can have direct effects on some preventive behaviours, such as when information about risky alcohol drinking prompts drinking at moderate levels or to stop drinking.

#### 2.2.5. Counsellor Training and Intervention Quality Assurance

The intervention assistant nurse counsellor delivered the interventions to men and women patients as per usual clinic services. The assistant nurse counsellors were trained to administer the intervention protocol through role playing and general skills training techniques in a 5 day workshop [30]. Site visits were done bi-weekly by the project manager to offer support and supervision to the trained assistant nurse counsellors. In addition, during implementation, assistant nurse counsellors were observed “ *in vivo*” for adherence to the detailed 15 steps counselling protocol by an external staff [30]. 

### 2.3. Measures

*Alcohol consumption:* The 10-item Alcohol Disorder Identification Test (AUDIT) [28] assesses alcohol consumption levels (three items), symptoms of alcohol dependence (three items), and problems associated with alcohol use (four items). Heavy episodic drinking is defined as the consumption of six standard drinks (10 g alcohol) or more on a single occasion [31]. In South Africa a standard drink is 12 g alcohol. Because AUDIT is reported to be less sensitive at identifying risk drinking in women [32], the cut-off points of binge drinking for women (4 units) were reduced by one unit as compared with men (5 units), as recommended by Freeborn *et al.* [32]. Responses to items on the AUDIT are rated on a 4-point Likert scale from 0 to 4, for a maximum score of 40 points. Higher AUDIT scores indicate more severe levels of risk; scores 8 or more indicate a tendency to problematic drinking or hazardous or harmful drinking, 9–19 hazardous and 20–40 harmful or probable dependent drinking [31]. To comply with the timeline of this study, all subjects will be asked for their alcohol consumption in the previous 6 months rather than 1 year. Cronbach alpha for the AUDIT was at baseline 0.75, 1st follow-up 0.80 and 2nd follow-up assessment 0.84. The between-group difference in the mean AUDIT score at follow-up was designated the primary outcome.

*Drinking norms* were assessed with the first two items of the Wing drinking norms questionnaire [33], e.g., “How often do you think the typical person your age, rank, and gender has a drink containing alcohol?” Response options ranged from 1 = never to 5 = 4 or more times a week. Cronbach alpha was 0.65 and 0.74 for the drinking norms index in this sample at Time 1 and Time 3, respectively.

Attitudes towards risk behaviour (alcohol). Participants were asked to give a rating on a 10-point scale from 1 = of very low importance to 10 = of very great importance for the relevance of “Not to drink too much alcohol.” 

*Tobacco use:* Two questions were asked about the use of tobacco products. (1) Do you currently use one or more of the following tobacco products (cigarettes, snuff, chewing tobacco, cigars, *etc.*)? Response options were “yes” or “no”. (2) In the past month, how often have you used one or more of the following tobacco products (cigarettes, snuff, chewing tobacco, cigars, *etc.*)? Response options were once or twice, weekly, almost daily and daily. *Cannabis use* was assessed with the question, “On how many days in the past 30 days have you used dagga (or cannabis)?” Current cannabis use was defined as any use in the past month.

Respondents rated their *health* with the question, “In general, would you say that your health is excellent, very good, good, fair, or poor?” This was dichotomized into (1) poor, fair or good and (2) very good or excellent.

*Post traumatic stress disorder* (PTSD). A 7-item screener was used to identify PTSD symptoms in the past month [34,35]. The introductory statement asked, “In your life, have you ever had any experience that was so frightening, horrible, or upsetting that, in the past month...” Items were (1) “Did you avoid being reminded of this experience by staying away from certain places, people, or activities?” (2) “Did you lose interest in activities that were once important or enjoyable?” (3) “Did you begin to feel more isolated and distant from other people?” (4) “Did you find it hard to have love or affection for other people?” (5) “Did you begin to feel that there was no point in planning for the future?” (6) “After this experience were you having more trouble than usual falling asleep or staying asleep?”and (7) “Did you become jumpy or get easily startled by ordinary noises or movements?”. Response options were “Yes”and “No”. Consistent with epidemiological evidence, participants who answered affirmatively to at least four of the questions were considered to have a positive screen for PTSD [34,35]. Cronbach alpha for the PTSD screen at Time 1 and at Time 3 in this sample were 0.70 and 0.78, respectively.

We assessed *depressive symptoms* using the 10-item version of the Centers for Epidemiologic Studies Depression Scale (CES-D) [36]. While the CES-D 10-item survey has not been directly compared to clinical diagnosis of major depression, the sensitivity and specificity of the CES-D 20-item survey has been reported to average 80% and 70%, respectively, compared to formal diagnostic interview [37]. In accordance with Andresen *et al.* [36], the possible range of the 10-item scale is 0 to 30, and a cut-off score of fifteen or higher indicates the presence of severe depressive symptoms. Cronbach alpha for the CES-D 10 at Time 1 and at Time 3 in this sample were 0.70 and 0.78, respectively.

We classified students according to whether they lived on or off campus accommodation. Socioeconomic background was assessed by rating their family background as wealthy (within the highest 25% in South Africa, in terms of wealth), quite well off (within the 50% to 75% range for their country), not very well off (within the 25% to 50% range for South Africa), or quite poor (within the lowest 25% in their country, in terms of wealth). We subsequently divided the students into poorer (not very well off and quite poor) and wealthier (wealthy, quite well off) categories. 

### 2.4. Data Analysis

Means, standard deviations, and percentages were used for descriptive statistics. T-test for continuous data and chi-square for categorical data were used to examine differences between groups. 

The primary outcome was measured at three time points: baseline, six and at 12 months. If a student dropped out, and is not present on the day of the interview or refuses to answer questions the primary outcome at the end point of the trial was missing. Therefore, except for the baseline measurement, no post-randomization information was available for these participants. The extent of the missing component was 3.3% at 12 months. The single follow-up measurement and the extent of the expected dropout was considered for the statistical method that was used to provide an unbiased estimate of the intervention effect under the principle of intention to treat. The method used to take account of the within-subject correlation resulting from successive observations, the repeated continuous or binary nature of the primary and secondary outcome and the missing data at follow-up is a generalized estimation equations (GEE) approach [38]. 

Randomization had been on the individual level, and to correct for baseline differences between the two groups, multilevel logistic regression was performed for binominal, and multilevel Poisson loglinear regression for continuous variables. Outcomes were calculated per follow up measurement and were adjusted for baseline gender and alcohol use. Possible effect modification was analyzed using interaction terms of these variables with randomization status. Estimated treatment effects are reported with 95% confidence intervals. Descriptive statistics were calculated for baseline and follow-up. IBM SPSS for Windows version 20.0 (SPSS, Inc., Chicago, IL, USA) was used for the calculations.

## 3. Results and Discussion

### 3.1. Screening and Randomization

**Figure 1:**
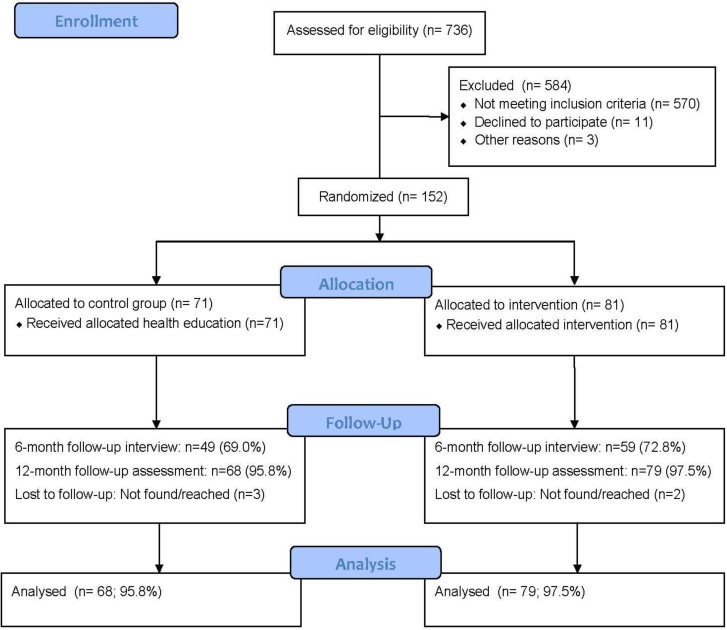
Flow-chart of participants in the trial.

Figure 1 summarizes student identification, recruitment, randomization, and follow-up numbers. We identified 736 university students, of which 570 screened negative for alcohol, 11 refused to participate and three were found ineligible, resulting in 152 university students who screened 8 or more on the AUDIT. Of the 722 screened for alcohol and agreed to participate in the trial 152 (21.1%) tested positive for the AUDIT (score 8 or more). Participants were individually randomized into 71 in the control and 81 in the intervention group. As illustrated in Figure 1, response rates were higher in the second compared to the first follow-up. At the 6-month follow-up, response rates for the control and intervention were 69% and 73%, respectively, and at 12 months, the control and intervention group response rates were 95.8% and 97.5%, respectively. In the control group 4.2% did not complete the last follow-up survey (*i.e.*, the dropout rate was 4.2%); in the intervention group, 2.5% did not complete the last follow-up survey. 

### 3.2. Brief Intervention Implementation Fidelity Analysis

About 10% of the brief intervention sessions were observed by external staff. In 82% of the intervention sessions, the counsellors implemented at least 13 of the 15 requisite intervention steps (including, (1) Establish AUDIT score, (2) Transitional statement, (3) Drinkers Pyramid, (4) Effects of high-risk drinking, (5) Discuss need to cut down or stop drinking, (6) Discuss sensible limits, (7) Review “What’s a standard drink” (8) Readiness ruler, (9) Good reasons for drinking less, (10) High-risk situations “Habit breaking plan”, (11) What to do when you are tempted, (12) People need people, (13) What to do about boredom, (14) Depression and (15) How to stick to your plans). 

### 3.3. Participant Characteristics

Table 1 summarizes and compares sociodemographic, health variables and alcohol-related characteristics of the study participants by different study groups. The study groups were equivalent on a all characteristics apart from AUDIT levels of alcohol use. Despite randomization, there was evidence of inequality between the control and the intervention group with regard to the severity of alcohol use. Overall, the study sample was 87.3% male, averaged 21.9 years of age (SD = 3.5), 39.7% were first year, 27.8% second year and 32.5% third or more year students and 77.6% were siding on the university campus.With regard to health variables, the overall mean score on the AUDIT was 16.2 (SD = 7.1), 72.4% were level 2 or 3 alcohol users (AUDIT scores 8–19), 27.6% level 4 (AUDIT scores 20–40), 23.7% were daily or almost daily tobacco users, and 29.5% were current (past month) cannabis users, 55.3% rated their health as very good or excellent, and university students reported to be suffering from PTSD (23.8%) and depression (12.6%) (see Table 1).

**Table 1:** Baseline characteristics stratified by study condition.

Variables	Control n = 71 (%)	Intervention n = 81 (%)	t/χ^2^	*P*-value
**Socio-demographic variables**				
Gender (N, % male)	65 (92.9)	66 (82.5)	χ^2 ^3.62	0.057
Age in years (M, SD)	22.1 (3.7)	21.7 (3.4)	t 0.67	0.503
*Year of study*				
First year	23 (39.7)	27 (39.7)	χ^2^ 0.17	0.920
Second year	17 (29.3)	18 (26.5)		
Third or more year	18 (31.0)	23 (33.8)		
*Family background*				
Wealthier (wealthy, quite well off)	32 (45.3)	34 (43.0)	χ^2 ^0.11	0.743
Poorer (not very well off or quite poor)	38 (54.3)	45 (57.0)		
*Residence*				
On campus	57 (80.3)	61 (75.3)	χ^2^ 0.54	0.463
Off campus	14 (19.7)	20 (24.7)		
**Health variables**				
Alcohol use (AUDIT score) (M,SD)	14.0 (6.1)	18.0 (7.3)	t −3.66	<0.001 ***
Drinking norms score (range 2–10)	7.2 (1.9)	7.1 (2.2)	t 0.35	0.729
Importance of not drinking too much Alcohol score (range 1–10)	7.1 (2.9)	7.0 (2.9)	t 0.18	0.858
Daily or almost daily tobacco use	16 (22.5)	20 (24.7)	χ^2 ^0.10	0.755
Past month cannabis use	18 (25.4)	27 (33.3)	χ^2 ^1.16	0.282
*Perceived health status*				
Poor/Fair/Good	33 (46.5)	35 (43.2)	χ^2 ^0.16	0.686
Very good/Excellent	38 (53.8)	46 (56.8)		
PTSD (4 or more items)	12 (17.6)	23 (29.1)	χ^2 ^2.65	0.104
Depression (15 or more scores)	5 (7.5)	12 (17.6)	χ^2 ^3.18	0.075

Notes: n (sample); M (Mean); SD (Standard deviation); t (t-value); χ^2^ (Chi-square) and *** *P* < 0.001.

### 3.4. Alcohol Use Outcomes

There were significant reductions in self-reported alcohol consumption (AUDIT total score and heavy episodic drinking) over time across treatment groups. Among the 147 (96.7%) university students who also attended the 12-month follow-up session, the intervention effect on the AUDIT score was −1.5, which was statistically significant (*P* = 0.004). Compared to the control group, the brief intervention group achieved a significant reduction of alcohol use. The intervention effect of the brief intervention was greater for the possible alcohol dependend group than for the high risk group. The mediator variable drinking norms reduced over time across treatment groups and “the importance of not drinking too much alcohol” increased over time across treatment groups, but this was not significant. Further, the depression scores marginally significantly decreased over time across treatment groups, while other substance use (tobacco and cannabis use), self-rated health status and PTSD scores did not change over time across treatment groups (see Table 2).

**Table 2:** Alcohol-related outcome measures at baseline, 6-month and 12-month follow-up.

Variables	Time	Control	Intervention	β (95% CI)	*P*-value
**Criterion variables**					
AUDIT total (M, SD)	Baseline	14.0 (6.1)	18.0 (7.3)	−0.18 (−0.30, −0.06)	0.004 **
	6 months	11.6 (7.7)	11.3 (7.6)		
	12 months	11.0 (6.4)	13.5 (7.3)		
AUDIT (8–19) High risk (n, %)	Baseline	59 (83.1)	51 (63.0)	−0.18 (−0.30, −0.06)	0.090
	6 months	23 (46.9)	28 (47.5)		
	12 months	43 (63.2)	48 (60.8)		
AUDIT (20–40) Alcohol dependence (n, %)	Baseline	12 (16.9)	30 (37.0)	−0.71 (−1.29, −0.13)	0.013 *
	6 months	10 (20.4)	11 (18.6)		
	12 months	6 (8.8)	16 (20.3)		
Heavy episodic drinking score (range 0–5) ^1^ (M, SD)	Baseline	1.7 (0.9)	2.2 (1.0)	−0.44 (−0.76, −0.12)	0.007 **
	6 months	1.8 (1.1)	1.7 (1.1)		
	12 months	1.8 (1.0)	1.9 (1.2)		
**Mediator variables**					
Drinking norms (M, SD)	Baseline	7.2 (1.9)	7.1 (2.2)	0.04 (−0.03, 0.12)	0.271
	12 months	7.1 (1.9)	6.6 (2.2)		
Importance of not drinking too much alcohol (M, SD)	Baseline	7.3 (2.8)	7.0 (2.8)	−0.01 (−0.10, 0.09)	0.886
	12 months	8.2 (2.3)	8.4 (2.6)		
**Other health variables**					
Daily or almost daily tobacco use (n, %)	Baseline	16 (22.5)	20 (24.7)	−0.24 (−0.88, 0.41)	0.469
	12 months	16 (23.5)	24 (30.4)		
Current (past month) cannabis use (n, %)	Baseline	18 (25.4)	27 (33.3)	−0.24 (−0.84, 0.37)	0.445
	12 months	20 (29.9)	25 (31.6)		
Self-rated health status (rated from 1–5, with 5 being the highest) (M, SD)	Baseline	3.6 (1.0)	3.6 (0.9)	−0.02 (−0.09, 0.04)	0.501
	12 months	3.6 (0.9)	3.7 (0.9)		
PTSD score (M, SD)	Baseline	1.8 (1.7)	2.4 (2.0)	−0.17 (−0.44, 0.10)	0.221
	12 months	1.6 (2.0)	1.7 (1.9)		
Depression score (M, SD)	Baseline	7.4 (4.2)	9.7 (6.5)	−0.07 (−0.14, 0.01)	0.074
	12 months	8.7 (6.5)	9.1 (5.5)		

^1^ Heavy episodic drinking was defined for men 5 or more and for women 4 or more drinks on one occasion; Notes: N (sample); % (percent); M (Mean); SD (Standard deviation); β (Beta coefficient); CI (Confidence Intervals) and ** *P* < 0.01; * *P* < 0.05.

## 4. Discussion

To our knowledge, this is the first randomized trial to evaluate the effectiveness of a brief intervention for hazardous and harmful alcohol use among university students in Africa. Self-reported outcome data suggest that brief intervention can help reduce levels of hazardous and harmful alcohol use in those students attending a brief intervention in South Africa. From baseline to 6- and 12-month follow-up, alcohol consumption declined significantly over time across treatment groups. Although, in general, all university students who were hazardous or harmful drinkers reduced alcohol consumption throughout the 12-months follow-up, the respondents who received the brief intervention showed a higher decline in AUDIT scores and heavy episodic drinking during the follow-up compared to the control group. These results are consistent with previous research showing that brief interventions can be effective [9,22,39] and that a decline in drinking rates in control groups seems to happen. 

The decline in drinking rates in the control group may be attributed to the intervention effect of alcohol screening/follow-up and provision of health education leaflet on sensible alcohol drinking. McCambridge and Kypri [40] reviewed that simply answering questions on drinking in brief intervention trials appears to alter subsequent self-reported behaviour. This potentially generates a bias by exposing non-intervention control groups to an integral component of the intervention. The effects of brief alcohol interventions may thus have been consistently under-estimated. Although statistically significant, the overall effects of the one session intervention were modest and were larger for heavier or harmful than hazardous drinkers (AUDIT scores 8–19), meaning that students continued drinking at risky levels. Similar findings were also found in a study among American College students [41]. Future studies using larger samples of heavier drinking university students are needed to further evaluate the possibility that the efficacy of brief alcohol interventions varies with higher rates of alcohol consumption [41].

Our study did not find mediator variables (alcohol risk attitudes and normative beliefs) to be associated with the alcohol treatment outcome. This is contrary to a review by Scott-Sheldon *et al.* [42] that found individual-level interventions for college students improved normative beliefs [42]. One explanation may be that attitudes and norms concerning alcohol’s effects are well established [43]. Brief interventions known to be effective in changing substance use may be effective in reducing other health-compromising behaviours such as tobacco or cannabis use, which was not found in this study. In a recent review, McCambridge and Jenkins [44] also found that brief alcohol interventions do not also reduce cigarette smoking, and it appears unlikely that there exist other important secondary effects. The brief alcohol intervention which included a section on identifying and managing depression had a marginal effect on the reduction of depressive symptoms in the intervention compared to the control group. Depression has been found to be associated with alcohol use [17,19,20]. This shows the importance of including a module on depression management in brief interventions for alcohol problems. 

## 5. Study Limitations

Our study has several limitations, including the loss of students at the first follow-up point and the circumstances of recruitment. Despite randomization there were baseline differences between the two groups on the main outcome measure (alcohol use). Although we controlled for these differences, we cannot exclude that there are additional unmeasured baseline differences that confound the effect, a fact that reduces internal validity of the study. Further, alcohol use was only assessed by self-report. The consensus in the research community that self-reported alcohol consumption was valid derives mainly from conclusions drawn from studies undertaken in treatment contexts [45]. It is not clear whether influences on the validity of self-report may be different in South Africa. Bias in alcohol consumption may have resulted from self-reported outcome measures. Future studies should consider to assess alcohol consumption by both self-report and objective measures, such as blood alcohol level. Finally, the study included volunteering students from only one specific public university and given the variability in drinking across educational settings, our results may not be generalizable to all other student populations in South Africa.

## 6. Conclusions

The study provides evidence of effective brief intervention by assistant nurses with hazardous and harmful drinkers in a university setting in South Africa. The short duration of the brief intervention makes it a realistic candidate for use in a university setting. Our findings also extend previous studies by determining that the brief alcohol intervention did not vary in efficacy based on student sociodemographic variables nor on health variables including posttraumatic stress, thus highlighting the implications for wide use of this intervention in university settings. Further, this is one of the very few studies in which severity of alcohol use did not influence intervention efficacy. Coupled with this, this brief alcohol intervention seems also effective among higher alcohol use groups, which has implications for developing future efforts within university contexsts.

## References

[1] World Health Organtization (WHO) (2011). Global Status Report on Alcohol and Health.

[2] Rehm J., Rehn N., Room R., Monteiro M., Gmel G., Jernigan D., Frick U. (2003). The global distribution of average volume of alcohol consumption and patterns of drinking. Eur. Addict. Res..

[3] Schneider M., Norman R., Parry C., Bradshawm D., Plüddemann A. (2007). South African comparison risk assessment collaborating group: Estimating the burden of disease attributable to alcohol use in South Africa in 2000. S. Afr. Med. J..

[4] Reid M.C., Fiellin D.A., O’Connor P.G. (1999). Harzardous and harmful alcohol consumption in primary care. Arch. Intern. Med..

[5] Zverev Y. (2008). Problem drinking among university students in Malawi. Coll. Anthropol..

[6] Young C., de Klerk V. (2008). Patterns of alcohol usage on a South African university campus: The findings of two annual drinking surveys. Afr. J. Drug Alcohol Stud..

[7] Young C., Mayson T. (2010). The Alcohol Use Disorders Identification Scale (AUDIT) normative scores for a multiracial sample of Rhodes University residence students. J. Child Adol. Men. Health.

[8] Alexandre E., Bowen A. (2004). Excessive drinking in college: Behavioral outcome, not binge, as a basis for prevention. Addict. Behav..

[9] Simão M.O., Kerr-Corrêa F., Smaira S.I., Trinca L.A., Floripes T.M., Dalben I., Martins R.A., Oliveira J.B., Cavariani M.B., Tucci A.M. (2008). Prevention of “risky” drinking among students at a Brazilian university. Alcohol Alcohol..

[10] Wicki M., Kuntsche E., Gmel G. (2010). Drinking at European universities? A review of students’ alcohol use. Addict. Behav..

[11] Borsari B., Carey K.B. (2001). Peer influences in college drinking: A review of the research. J. Subst. Abuse..

[12] Deressa W., Azazh A. (2011). Substance use and its predictors among undergraduate medical students of Addis Ababa University in Ethiopia. BMC Public Health..

[13] John B., Alwyn T. (2010). Alcohol Related Social Norm Perceptions in University Students: Effective Interventions for Change. http://alcoholresearchuk.org/downloads/finalReports/AERC_FinalReport_0072.pdf.

[14] Steyl T., Phillips J. (2011). Actual and perceived substance use of health science students at a university in the Western Cape, South Africa. Afr. Health Sci..

[15] Utpala-Kumar R., Deane F.P. (2012). Heavy episodic drinking among university students: Drinking status and perceived normative comparisons. Subst. Use Misuse..

[16] Vantamay S. (2009). Alcohol consumption among university students: Applying a social ecological approach for multi-level preventions. Southeast Asian J. Trop. Med. Publ. Health.

[17] Adewuya A.O., Ola B.A., Aloba O.O., Mapayi B.M., Oginni O.O. (2006). Depression amongst Nigerian university students. Prevalence and sociodemographic correlates. Soc. Psychiatry Psychiatr. Epidemiol..

[18] Atwoli L., Mungla P.A., Ndung’u M.N., Kinoti K.C., Ogot E.M. (2011). Prevalence of substance use among college students in Eldoret, Western Kenya. BMC Psychiatry.

[19] O’Donnell K., Wardle J., Dantzer C., Steptoe A. (2006). Alcohol consumption and symptoms of depression in young adults from 20 countries. J. Stud. Alcohol..

[20] Peltzer K. (2003). Depressive symptoms in relation to alcohol and tobacco use in South African University students. Psychol. Rep..

[21] Bachrach R.L., Read J.P. (2012). The role of posttraumatic stress and problem alcohol involvement in university academic performance. J. Clin. Psychol..

[22] Carey K.B., Scott-Sheldon L.A., Carey M.P., DeMartini K.S. (2007). Individual-level interventions to reduce college student drinking: A meta-analytic review. Addict. Behav..

[23] Seigers D.K., Carey K.B. (2011). Screening and brief interventions for alcohol use in college health centers: A review. J. Am. Coll. Health.

[24] (2012). The Physicians’ Guide to Helping Patients with Alcohol Problems.

[25] Pensuksan W.C., Taneepanichskul S., Williams M.A. (2010). A peer-drinking group motivational intervention among Thai male undergraduate students. Int. J. Drug Policy.

[26] Babor T.F., Higgins-Biddle J.C. (2001). Brief Intervention for Hazardous and Harmful Drinking. A Manual for Use in Primary Care Settings.

[27] Fisher W.A., Fisher J.D., Harman J., Suls J., Wallston K. (2003). The information-motivation-behavioural skills model: A general social psychological approach to understanding and promoting health behavior. Social Psychological Foundations of Health and Illness.

[28] Raistrick D., Heather N., Godfrey C. (2006). Review of the Effectiveness of Treatment for Alcohol Problems.

[29] Raistrick D., Tober G. (2004). Psychosocial interventions. Psychiatry.

[30] Pengpid S., Peltzer K., Skaal L., van der Heever H., van Hal G. (2012). Screening and brief intervention for alcohol problems in Dr George Mukhari Hospital out-patients in Gauteng, South Africa: A single-blinded randomized controlled trial protocol. BMC Public Health.

[31] Babor T.F., Higgins-Biddle J.C., Saunders J.B., Monteiro M.G. (2001). AUDIT: The Alcohol Use Disorders Identification Test. Guidelines for Use in Primary Care.

[32] Freeborn D.K., Polen M.R., Hollis J.F., Senft R.A. (2000). Screening and brief intervention for hazardous drinking in an HMO: Effects on medical care utilization. J. Behav. Health Serv. Res..

[33] Wing K. (2010). Drinking Norms Questionnaire. http://www.afcrossroads.com/Drinking_Norms_Questionnaire2.pdf.

[34] Kimerling R., Ouimette P., Prins A., Nisco P., Lawler C., Cronkite R., Moos R.H. (2006). Brief report: Utility of a short screening scale for DSM-IV PTSD in primary care. J. Gen. Intern. Med..

[35] Sikkema K.J., Watt M.H., Meade C.S., Ranby K.W., Kalichman S.C., Skinner D., Pieterse D. (2011). Mental health and HIV sexual risk behavior among patrons of alcohol serving venues in Cape Town, South Africa. J. Acquir. Immune Defic. Syndr..

[36] Andresen E.M., Malmgren J.A., Carter W.B., Patrick D.L. (1994). Screening for depression in well older adults: Evaluation of a short form of the CES-D (Center for Epidemiologic Studies Depression Scale). Am. J. Prev. Med..

[37] Mulrow C.D., Williams J.W., Gerety M.B., Ramirez G., Montiel O.M., Kerber C. (1995). Case-finding instruments for depression in primary care settings. Ann. Intern. Med..

[38] Molenberghs G., Kenward M.G. (2007). Missing Data in Clinical Studies.

[39] Baer J.S., Kivlahan D.R., Blume A.W., McKnight P., Marlatt G.A. (2001). Brief intervention for heavy-drinking college students: 4-year follow-up and natural history. Am. J. Public Health..

[40] McCambridge J., Kypri K. (2011). Can simply answering research questions change behaviour? Systematic review and meta analyses of brief alcohol intervention trials. PLoS One.

[41] Murphy J.G., Duchnick J.J., Vuchinich R.E., Davison J.W., Karg R.S., Olson A.M., Smith A.F., Coffey T.T. (2001). Relative efficacy of a brief motivational intervention for college student drinkers. Psychol. Addict. Behav..

[42] Scott-Sheldon L.J., Demartini K.S., Carey K.B., Carey M.P. (2009). Alcohol interventions for college students improves antecedents of behavioural change: Results from a meta-analysis of 34 randomized controlled trials. J. Soc. Clin. Psychol..

[43] Borsari B., Carey K.B. (2000). Effects of a brief motivational intervention with college student drinkers. J. Consult. Clin. Psychol..

[44] McCambridge J., Jenkins R.J. (2008). Do brief interventions which target alcohol consumption also reduce cigarette smoking? Systematic review and meta-analysis. Drug Alcohol Depend..

[45] Noknoy S., Rangsin R., Saengcharnchai P., Tantibhaedhyangkul U., McCambridge J. (2010). RCT of effectiveness of motivational enhancement therapy delivered by nurses for hazardous drinkers in primary care units in Thailand. Alcohol Alcohol..

